# Spastic Paraparesis After SARS-CoV-2 Infection Without Radiological Changes

**DOI:** 10.7759/cureus.23054

**Published:** 2022-03-11

**Authors:** Sanela Zukic, Ena Topcic, Renata Hodzic, Osman Sinanovic, Mirjana Vidovic

**Affiliations:** 1 Department of Neurology, University Clinical Center Tuzla, Tuzla, BIH; 2 Department for Physical Medicine and Rehabilitation, University Clinical Center Tuzla, Tuzla, BIH; 3 Neurology, School of Medicine, University of Tuzla, Tuzla, BIH

**Keywords:** immune-related neurological complications, immune-mediated spastic paraparesis, htlv-1 spastic paraparesis, mri negative spastic paraparesis, spastic paraparesis after covid – 19, sars cov-2 infection, spastic paraparesis

## Abstract

Coronavirus disease 2019 (COVID-19) is primarily a disease of the respiratory system but severe acute respiratory syndrome coronavirus 2 (SARS-CoV-2) may cause several immune-related complications including different neurological disorders, such as myelopathy with paraparesis.In this atypical case a female patient with progressive spastic paraparesis after COVID-19 infection, brisk reflexes and positive Babinski sign, reduced vibratory sensation to the thoracic level, elevated immunoglobulin levels (IgG) in cerebrospinal fluid, but negative magnetic resonance imaging (MRI) of the brain and spine, is presented.

A 57-year-old woman with spastic paraparesis and inability to walk was admitted to our neurological department. About four months before hospitalization, she started feeling numbness and tingling in the feet and lumbar spine area. Gradually, numbness and tingling ascended to the thoracic spine level Th7/8, and she developed weakness mostly in her legs. In the neurological exam she had spastic paraparesis. MRI of the brain, cervical and thoracic spine did not reveal any signal abnormality. Serological testing for SARS-CoV-2 was performed and results were highly positive IgG and IgM+IgA levels. The lumbar puncture finding confirmed the suspicion of immune-related complications after SARS-CoV-2 infection (intrathecal IgG synthesis).

This case draws attention to spastic paraparesis or progressive MRI-negative myelitis after SARS-CoV-2 infection, which obviously has immune-mediated pathogenesis that happen in response to the virus or its antibodies. Similarities in spastic paraparesis after human T-lymphotropic virus (HTLV-1) or human immunodeficiency virus (HIV-1) and SARS-CoV-2 infections were observed. The patient had a good response to corticosteroid therapy and had good recovery.

## Introduction

Coronavirus disease 2019 (COVID-19) is primarily a disease of the respiratory system but severe acute respiratory syndrome coronavirus 2 (SARS-CoV-2) may cause several immune-related complications, including different neurological disorders such as myelopathy with paraparesis. This type of spastic paraparesis after COVID-19 infection has been described in patients with human T-cell lymphotropic virus (HTLV-1) infection - acute spastic paraparesis that results in painful stiffness and weakness of the legs [1]. The typical presentation is a slowly progressive and non-compressive paraparesis in the lower limbs, widespread pyramidal signs, urinary urgency or incontinence, constipation, and subtle objective sensory signs (mainly decreased vibration sense) [2]. In this atypical case, a female patient with progressive spastic paraparesis after COVID-19 infection but negative magnetic resonance imaging (MRI) of the brain and spine is presented.

## Case presentation

A 57-year-old woman with spastic paraparesis and inability to walk was admitted to our neurological department. About four months before hospitalization, she started feeling numbness and tingling in the feet and lumbar spine area. Gradually, numbness and tingling ascended to the thoracic spine level Th7/8 and she developed weakness mostly in her legs. At the same time bladder (retention) and bowel dysfunction (constipation) started. She visited a neurologist as an outpatient, and she was recommended for MRI of the brain and spine, which did not reveal any signal abnormality.

In the neurological exam she had normal upper extremity strength, but she was only able to move the legs on the ground, with a grade of 2 muscle strength on the Manual Muscle Testing scale. She had very brisk reflexes on the legs with clonus phenomenon at the ankle and increased muscle spasms. Babinski sign was positive on both sides. There was reduced vibratory sensation to the thoracic spine Th7 level, and she described the phenomenon as a painful "hoop" around the thoracic spine. A urinary catheter was inserted. In our hospital, the MRI of cervical and thoracic spine (1.5 T with and without contrast) was also reported to be normal. Laboratory results such as complete blood count, glucose, sodium, potassium, electrolytes, liver panel, copper, B12, and vitamin D were normal. Autoimmune laboratory tests were negative (immunoglobulins IgA, IgG, IgM; antinuclear antibodies [ANA]; and antineutrophil cytoplasmic antibodies [c-ANCA and p-ANCA]). Electromyoneurography did not show any peripheral etiology for her symptoms. A SARS-CoV-2 nasopharyngeal polymerase chain reaction (PCR) test done at this time was negative, but serological testing was performed and results were highly positive IgG (23.09) and IgM+IgA (12.99). Patient later recalled that two months before the onset of neurological symptoms, she had a cold-like illness. The lumbar puncture finding confirmed the suspicion of immune-related complications after SARS-CoV-2 infection (intrathecal IgG synthesis). The cerebrospinal fluid analysis showed elevated IgG (0.4). Extensive cerebrospinal fluid (CSF) panel was negative for IgM.

The patient received a high dose of pulse intravenous steroid (methylprednisolone 1000 mg i.v. for three days) with immediate improvement of symptoms; she felt less pain and more strength in the legs. The patient was transferred to rehabilitation with a prescription of oral prednisone 60 mg daily for 12 weeks followed by a slow taper to 40 mg. She was also treated with physical therapy; physiotherapy, transcutaneous electrical nerve stimulation (TENS) and magnetotherapy. During the physical treatment, improvement was achieved in muscle strength and mobility of the lower extremities, improvement of balance in sitting position, and after two months she was able to walk with bilateral assistance. The Barthel Index improved from 12 to 55 (independency in feeding 5/8; moving from wheelchair to bed and return 0/8; grooming 3/5; transferring to and from a toilet 0/5; bathing 0/3; walking on a level surface 0/3; going up and down stairs 0/0; dressing 2/8; and continence of bowels 2/5 and bladder 0/10). On the Functional Independence Measure (FIM), improvement was from 49 to 83.

## Discussion

The most common neurological complications described in the literature after and during SARS-CoV-2 infection are headache, anosmia, encephalitis, delirium, cerebrovascular diseases, myelitis, Guillain-Barre syndrome, etc. [3-5]. Here progressive spastic paraparesis after COVID-19 infection, with negative MRI of the brain and spine, is presented.

This type of spastic paraparesis after COVID-19 infection was described in patients with HTLV-1 and HIV infection; acute spastic paraparesis which results in painful stiffness and weakness of the legs. The presentation is with a slow progressive and non-compressive paraparesis in the lower limbs, widespread pyramidal signs, urinary urgency or incontinence, constipation, and subtle objective sensory signs (mainly decreased vibration sense) [2]. In that light, Kliger and Levanon [6] described a cloaked similarity between HIV-1 and SARS-CoV agents. It is known that viral envelope protein mediates the membrane fusion process between viral and cellular membrane Gp41 in the retrovirus HIV-1, and S2 in the coronavirus SARS-CoV is responsible for viral-induced membrane fusion, and a surface subunit that is responsible for the interaction with the cellular receptors. Although there is no sequence homology between the SARS-CoV S2 and HIV-1 gp41, comprehensive sequence analysis reveals that all the above-mentioned elements of gp41 are present also in S2. The transmembrane glycoprotein (Gp41) and surface glycoprotein, binds to CD4 receptors on the surface of CD4+ cells. SARS-CoV S2 and gp41 share the same two α helices, suggesting that the two viruses could follow an analogous membrane fusion mechanism. The proposed mechanism by which SARS-CoV-2 infection may result in immune system dysfunction is not fully understood. SARS-CoV-2 spike glycoprotein (S) directly binds to the CD4 molecule, which in turn mediates the entry of SARS-CoV-2 in T helper cells in a mechanism that also requires ACE2 and TMPRSS2. CD4-mediated SARS-CoV-2 infection of T helper cells may explain the poor adaptive immune response of many COVID-19 patients [7,8].

This atypical presentation of spastic paraparesis after SARS-CoV-2 infection has very similar immune-mediated pathogenesis that happened in response to the virus or its antibodies, so the concept of autoimmune mechanisms has an important role in the interpretation of pathogenesis. Although the pathogenesis of neuronal retroviral damage remains unclear, the hypothesis is that the virus causes chronic inflammation in the central nervous system and excessive and strong immune response [9]. Moreover, the levels of antibodies against SARS-CoV-2 decay rapidly after recovery, suggesting that SARS-CoV-2 infection may exert profound and long-lasting complications to adaptive immunity [10]. In literature, we found cases with neurological complications after COVID-19 illness, such as similar rapidly progressive paraparesis but MRI showed multifocal myelitis affecting the cervical cord [2], or spinal cord dysfunction in patients with three different changes on MRI; ischemia, spinal epidural abscess and infection of Staphylococcus aureus [11]. In this case of paraparesis MRI repeatedly remained normal, so analyzing the mechanism of disease we can assume the immune-mediated pathogenesis in response to the virus or its antibodies. The immune response to SARS-CoV-2 is a double-edged sword; the immune cells that are recruited to the infection site can inflict further damage to the infected tissues [12]. We treated the patient with corticosteroid therapy, since its currently the most widely accepted treatment for HTLV-1 associated myelopathy [13].

## Conclusions

This case draws attention to spastic paraparesis or progressive MRI-negative myelitis after SARS-CoV-2 infection, which obviously has immune-mediated pathogenesis that happens in response to the virus or its antibodies. Similarities in spastic paraparesis after HTLV-1 (HIV-1) and SARS-CoV-2 infections were observed. The patient had a good response to corticosteroid therapy and had good recovery. 
